# Numerical Bifurcation Theory for High-Dimensional Neural Models

**DOI:** 10.1186/2190-8567-4-13

**Published:** 2014-07-25

**Authors:** Carlo R Laing

**Affiliations:** Institute of Natural and Mathematical Sciences, Massey University, Private Bag 102-904 NSMC, Auckland, New Zealand

**Keywords:** Pseudo-arclength, Continuation, Bifurcation, Neural field

## Abstract

Numerical bifurcation theory involves finding and then following certain types of solutions of differential equations as parameters are varied, and determining whether they undergo any bifurcations (qualitative changes in behaviour). The primary technique for doing this is numerical continuation, where the solution of interest satisfies a parametrised set of algebraic equations, and branches of solutions are followed as the parameter is varied. An effective way to do this is with pseudo-arclength continuation. We give an introduction to pseudo-arclength continuation and then demonstrate its use in investigating the behaviour of a number of models from the field of computational neuroscience. The models we consider are high dimensional, as they result from the discretisation of neural field models—nonlocal differential equations used to model macroscopic pattern formation in the cortex. We consider both stationary and moving patterns in one spatial dimension, and then translating patterns in two spatial dimensions. A variety of results from the literature are discussed, and a number of extensions of the technique are given.

**Electronic supplementary material:**

The online version of this article (doi:10.1186/2190-8567-4-13) contains supplementary material, which is available to authorized users.

## 1 Introduction

Many models that arise in computational neuroscience are of the form

1 $$\frac{\partial u}{\partial t}=g(u;\mu ),$$

where *u* may be a finite-dimensional vector or a function (of space and time, for example), *μ* is a vector of parameters, and the derivative is with respect to time, *t*. An ambitious goal when studying a model of the form (1) is to completely understand the nature of all of its solutions, for all possible values of *μ*. Analytically solving (1) would give us this information, but for many functions *g* such a solution is impossible to find. Instead we must concentrate on finding *qualitative* information about the solutions of (1), and on how they change as the parameter *μ* is varied. Questions we would like to answer include (a) What do typical solutions do as t→∞, i.e. what is the “steady state” behaviour of a neuron or neural system? (b) Are there “special” initial conditions or states that the system can be in for which the long time behaviour is different from that for a “typical” initial condition? (c) How do the answers to these questions depend on the values of *μ*? More particularly, can the dynamics of (1) change qualitatively if a parameter (for example, input current to a neuron) is changed? In order to try to answer these questions we often concentrate on certain types of solutions of (1). Examples include fixed points (i.e. values of *u* such that g(u;μ)=0), periodic orbits (i.e. solutions which are periodic in time), and (in spatially dependent systems) patterned states such as plane and spiral waves. Solutions like these can be stable (i.e. all initial conditions in some neighbourhood of these solutions are attracted to them) or unstable (some nearby initial conditions leave a neighbourhood of them).

For many systems of interest, finding solutions of the type just mentioned, and their stability, can only be done numerically using a computer. Even the simplest case of finding all fixed points can be non-trivial, as there may be many or even an infinite number of them. When *u* is high dimensional, for example when (1) arises as the result of discretising a partial differential equation such as the cable equation on a dendrite or axon [1, 2], finding the linearisation about a fixed point, as needed to determine stability, may also be computationally challenging.

In the simplest case, that of finding fixed points of (1) and their dependence on *μ*, there are two main techniques. The first is integration in time of (1) from a given initial condition until a steady state is reached. Then *μ* is changed slightly, the process is repeated, and so on. This is conceptually simple, and many accurate integration algorithms exist, but it has several disadvantages:

- There may be a long transient before a steady state is reached, requiring long time simulations.
- Only *stable* fixed points can be found using this method.
- For fixed *μ* there may be multiple stable fixed points, and which one is found depends on the value of the initial condition, in a way that may not be obvious.

The second technique for finding fixed points of (1) and their dependence on *μ*, which is the subject of this review, involves solving the algebraic equation g(u;μ)=0 directly. The stability of a fixed point is then determined by the linearisation of *g* about it, involving partial derivatives of *g*, rather than by time integration of (1).

Numerical bifurcation theory involves (among other things) finding fixed points and periodic orbits of models such as (1) by solving a set of algebraic equations, determining the stability of these solutions, and following them as parameters are varied, on a computer. The field is well developed, with a number of books [3–6], book chapters [7], reports [8] and software packages available [9–14]. The point of this article is not to cover numerical bifurcation theory in general, but to demonstrate and review its use in the study of some models from the field of computational neuroscience, in particular those that are high dimensional, resulting from the discretisation of systems of nonlocal differential equations commonly employed as large-scale models of neural tissue, such as the Wilson–Cowan [15] and Amari [16] models. We start in Sect. 2 by explaining the pseudo-arclength continuation algorithm and show how it can be applied to a simple model. Section 3 considers both stationary and moving patterns in one spatial dimension, while Sect. 4 gives an example of a pattern in two spatial dimensions. We discuss a number of extensions in Sect. 5 and conclude in Sect. 6.

## 2 A Low-Dimensional Model

**Fig. 1:**
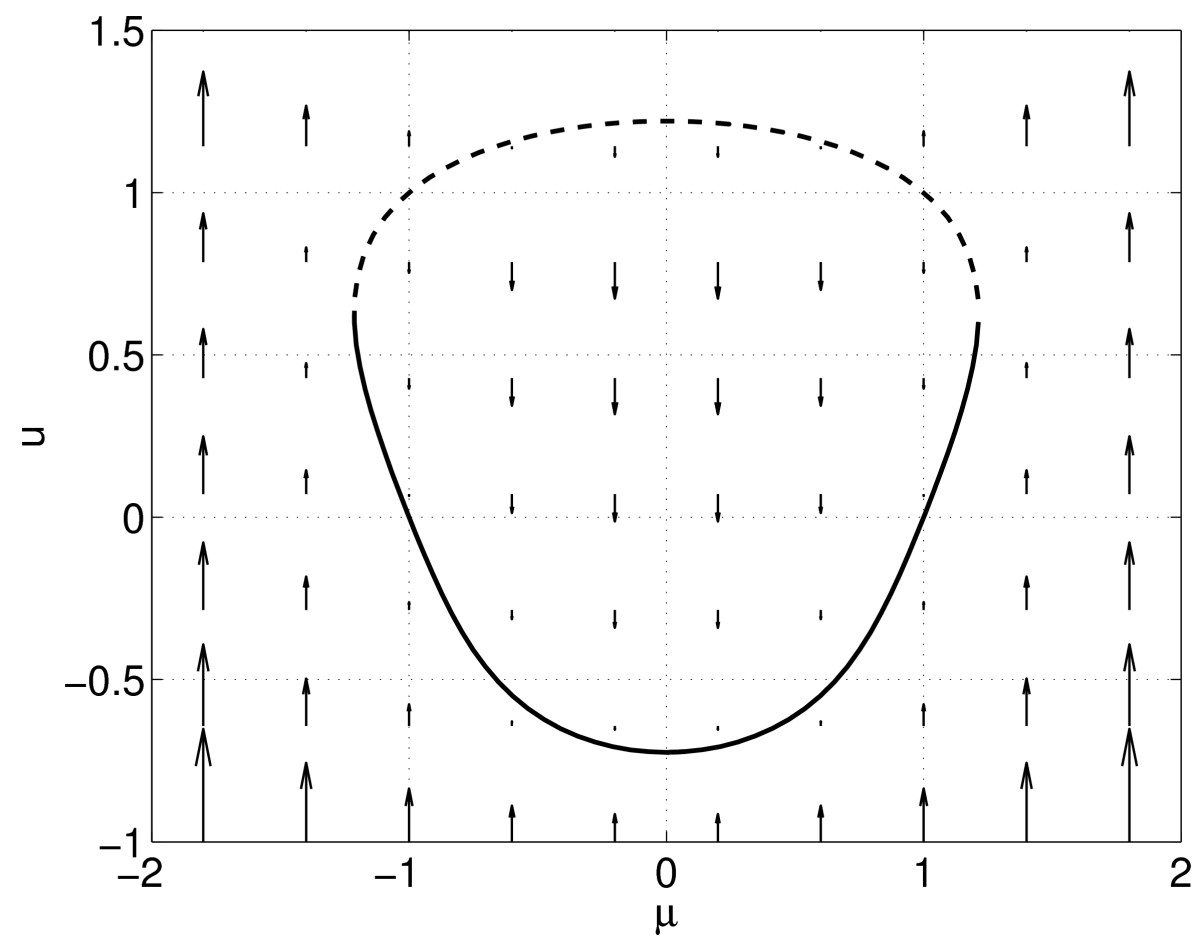
Dynamics of (2). The curve of stable fixed points is shown *solid*, while the unstable fixed points are shown *dashed*. *Arrows* show the direction of solutions for fixed *μ*

As a toy model we consider the differential equation

2 $$\frac{du}{dt}=g(u;\mu )\equiv u^{4}-u+\mu^{2}-1;\;u\in R,\mu \in R,$$

and assume that we cannot solve it analytically. We see that for *μ* large and positive, or large and negative, there are no fixed points, as du/dt is always positive. However, for small enough *μ* there may be fixed points. A full qualitative description of the dynamics of (2) is shown in Fig. 1 and we see that for *μ* small enough there are two fixed points, one stable and one unstable. We can find the stable fixed point by numerically integrating (2) forwards for a long time. (Note that if we chose the initial condition to be too large, this would not work.) We can also find this fixed point using Newton’s method, starting sufficiently close to it. Newton’s method has the advantage that its convergence does not depend on the stability of the fixed point, and thus it could also be used to find the unstable fixed point, if we started sufficiently close to it. (Clearly, numerical integration on its own is of little use if we want to find unstable objects.) The basic idea of numerical continuation is to take a fixed point, at one value of the parameter, and follow this solution as a parameter is varied, eventually tracing out, for example, the closed curve in Fig. 1. We would also like to know the stability of points on this curve, and that can be calculated as the curve is traced out, or afterwards.

**Fig. 2:**
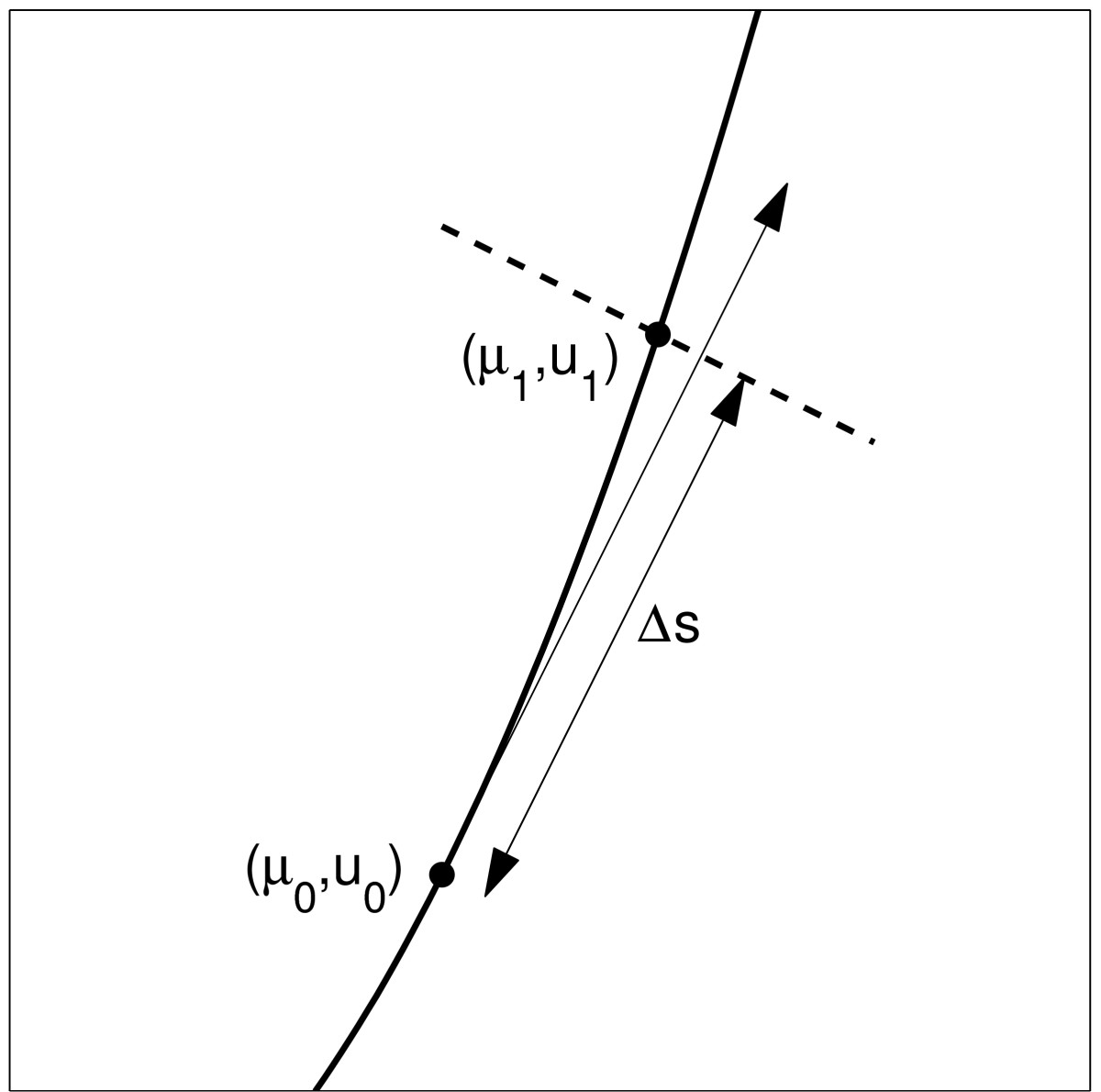
A schematic showing the relationship expressed by (3). The curve of solutions is *solid*, and the tangent to this curve at $\left(\mu_{0},u_{0}\right)$ is shown by the *single-headed arrow*. The *dashed line* is at right angles to this tangent

The method we use is pseudo-arclength continuation; other methods exist [4]. Given a point $\left(\mu_{0},u_{0}\right)$ on the curve shown in Fig. 1 (i.e. for which $g(u_{0};\mu_{0})=0$) we want to find a nearby point $\left(\mu_{1},u_{1}\right)$ which satisfies $g(u_{1};\mu_{1})=0$, i.e. also lies on the curve. We specify this point by saying that not only must it satisfy $g(u_{1};\mu_{1})=0$, but also that

3 $$(u_{1}-u_{0}){\dot{u}}_{0}+(\mu_{1}-\mu_{0}){\dot{\mu }}_{0}-\Delta s=0,$$

where Δ*s* is the pseudo-arclength stepsize chosen and $\left({\dot{u}}_{0},{\dot{\mu }}_{0}\right)$ is the tangent vector to the curve at $\left(\mu_{0},u_{0}\right)$, normalised to have length 1. The overdot indicates differentiation with respect to arclength, *s*. Geometrically, condition (3) states that $\left(\mu_{1},u_{1}\right)$ lies on the line perpendicular to the vector $\left({\dot{u}}_{0},{\dot{\mu }}_{0}\right)$, which at its closest point is a distance Δ*s* from $\left(u_{0},\mu_{0}\right)$. See Fig. 2 for a schematic.

The pseudo-arclength stepsize Δ*s* is chosen by the user, and as with many numerical schemes there is a tradeoff. Using a small stepsize will result in better coverage of the curve and faster convergence of Newton’s method (below), but at the expense of more calculations. To obtain $\left({\dot{u}}_{0},{\dot{\mu }}_{0}\right)$ we differentiate g(u;μ)=0 with respect to arclength and then evaluate at $\left(\mu_{0},u_{0}\right)$:

4 $$g_{u}{|}_{\left(\mu_{0},u_{0}\right)}{\dot{u}}_{0}+g_{\mu }{|}_{\left(\mu_{0},u_{0}\right)}{\dot{\mu }}_{0}=0,$$

where the subscripts indicate partial derivatives. A solution of this is then normalised. To find $\left(\mu_{1},u_{1}\right)$ we solve $g(u_{1};\mu_{1})=0$ and (3) simultaneously using the following Newton iteration:

5 $$(\begin{array}{c} u_{1}^{\left(i\right)}\\ \mu_{1}^{\left(i\right)} \end{array})=(\begin{array}{c} u_{1}^{\left(i-1\right)}\\ \mu_{1}^{\left(i-1\right)} \end{array})-J_{\left(i-1\right)}^{-1}(\begin{array}{c} g(u_{1}^{\left(i-1\right)},\mu_{1}^{\left(i-1\right)})\\ (u_{1}^{\left(i-1\right)}-u_{0}){\dot{u}}_{0}+(\mu_{1}^{\left(i-1\right)}-\mu_{0}){\dot{\mu }}_{0}-\Delta s \end{array}),$$

for $i=1,2,\ldots ,N_{N}$, where

6 $$J_{\left(i\right)}=(\begin{array}{cc} g_{u} & g_{\mu }\\ {\dot{u}}_{0} & {\dot{\mu }}_{0} \end{array}),$$

and the partial derivatives are evaluated at $\left(\mu_{1}^{\left(i\right)},u_{1}^{\left(i\right)}\right)$. We take $N_{N}$ Newton iterations and (assuming that (5) has converged) set $\left(\mu_{1},u_{1})=(\mu_{1}^{\left(N_{N}\right)},u_{1}^{\left(N_{N}\right)}\right)$. As an initial condition we can take $\left(\mu_{1}^{\left(0\right)},u_{1}^{\left(0\right)})=(\mu_{0}+{\dot{\mu }}_{0}\Delta s,u_{0}+{\dot{u}}_{0}\Delta s\right)$, i.e. the point where the tangent line meets the dashed line in Fig. 2. This point can be regarded as the result of a linear *prediction*, and Newton’s method (5) regarded as a *corrector* of this prediction. The stability of the fixed point $\left(\mu_{1},u_{1}\right)$ depends on the sign of $g_{u}$ evaluated at this point, and this has already been calculated as the top left entry in the Jacobian *J*.

To find the next point along the curve, $\left(\mu_{2},u_{2}\right)$ we use the Newton iteration

7 $$(\begin{array}{c} u_{2}^{\left(i\right)}\\ \mu_{2}^{\left(i\right)} \end{array})=(\begin{array}{c} u_{2}^{\left(i-1\right)}\\ \mu_{2}^{\left(i-1\right)} \end{array})-J_{\left(i-1\right)}^{-1}(\begin{array}{c} g(u_{2}^{\left(i-1\right)},\mu_{2}^{\left(i-1\right)})\\ (u_{2}^{\left(i-1\right)}-u_{1}){\dot{u}}_{1}+(\mu_{2}^{\left(i-1\right)}-\mu_{1}){\dot{\mu }}_{1}-\Delta s \end{array}),$$

for $i=1,2,\ldots ,N_{N}$, where the Jacobian is given by (6) and the partial derivatives are evaluated at $\left(\mu_{2}^{\left(i\right)},u_{2}^{\left(i\right)}\right)$. The tangent vector $\left({\dot{u}}_{1},{\dot{\mu }}_{1}\right)$ can be calculated using the analogue of (4), or by using the approximation

8 $${\dot{u}}_{1}\approx \frac{u_{1}-u_{0}}{\Delta s};\;{\dot{\mu }}_{1}\approx \frac{\mu_{1}-\mu_{0}}{\Delta s}.$$

This process can then be continued to find as many points on the curve as required. Note the following points:

- Pseudo-arclength continuation follows a curve of solutions in parameter/state space and can follow such a curve through a saddle-node bifurcation, even though such bifurcations can be thought of as involving the annihilation of two solutions. This is its main advantage over natural parameter continuation, for example, which fails at such a point [4, 17].
- Consider the structure of the equations being solved in (5). The first equation is g(u,μ)=0, and the last is the pseudo-arclength condition. This structure will be repeated in following sections.
- As presented, this method will find points in one direction along the curve given by g(u,μ)=0. If points in the other direction are required, simply replace the tangent vector by its negative, i.e. $\left({\dot{u}}_{0},{\dot{\mu }}_{0})\mapsto (-{\dot{u}}_{0},-{\dot{\mu }}_{0}\right)$ when calculating $\left(\mu_{1},u_{1}\right)$, and then continue as above.
- A given problem may have fixed points that lie on a closed curve, as in Fig. 1, or on an unbounded curve.
- There are a number of refinements that could be made to this algorithm to increase its efficiency. For example, one could terminate the Newton iterations once a solution has been found to within some accuracy, rather than after a fixed number of iterations [4]. One could also adapt the stepsize as the solution curve is traced out to avoid unnecessary iterations of Newton’s method [4, 17]. Another refinement is that if *u* and *μ* are of very different magnitudes it may be beneficial to scale one of them. One way to do this is to replace (3) by
  9 $$\theta^{2}(u_{1}-u_{0}){\dot{u}}_{0}+(\mu_{1}-\mu_{0}){\dot{\mu }}_{0}-\Delta s=0,$$
  where 0<θ≪1 if typical values of *u* are much larger than those of *μ*, and 1≪θ if the opposite is true.
- The algorithm above involves two nested loops. The inner loop finds a point on the curve of solutions, and the outer one steps along the curve.

The interested reader is encouraged to reproduce Fig. 1 using the method outlined above, and then to explore further. (See software at http://www.massey.ac.nz/~crlaing/code.htm.)

## 3 One-Dimensional Models

We now consider several types of pattern that occur in neural field models in one spatial dimension. Such models are used to study macroscopic pattern formation in the cortex, and take the form of nonlocal differential equations. For more background on such models see [16, 18–22], and the recent review [23]. We first consider stationary patterns.

### 3.1 Stationary Patterns

Consider a typical neural field model on a circular domain:

10 $$\frac{\partial u(x,t)}{\partial t}=-u(x,t)+\int_{-\pi }^{\pi }w(x-y)f\left(u(y,t)-h\right)\;dy,$$

where *w* is an even function, and

11 $$f(u)=\frac{1}{1+e^{-\beta u}}$$

is a sigmoidal function, where β>0 is a steepness parameter. The variable u(x,t) is the neural field at position *x* and time *t* and represents the activity of a population of neurons at that point. The function w(x−y) represents how neurons at position *y* affect those at position *x*, i.e. the network’s connectivity. Its evenness is a manifestation of the isotropy of the domain, i.e. that there is no preferred direction around the domain. The function *f* is referred to as the firing rate function, converting activity, *u*, to firing frequency, f(u), and *h* is a firing threshold.

**Fig. 3:**
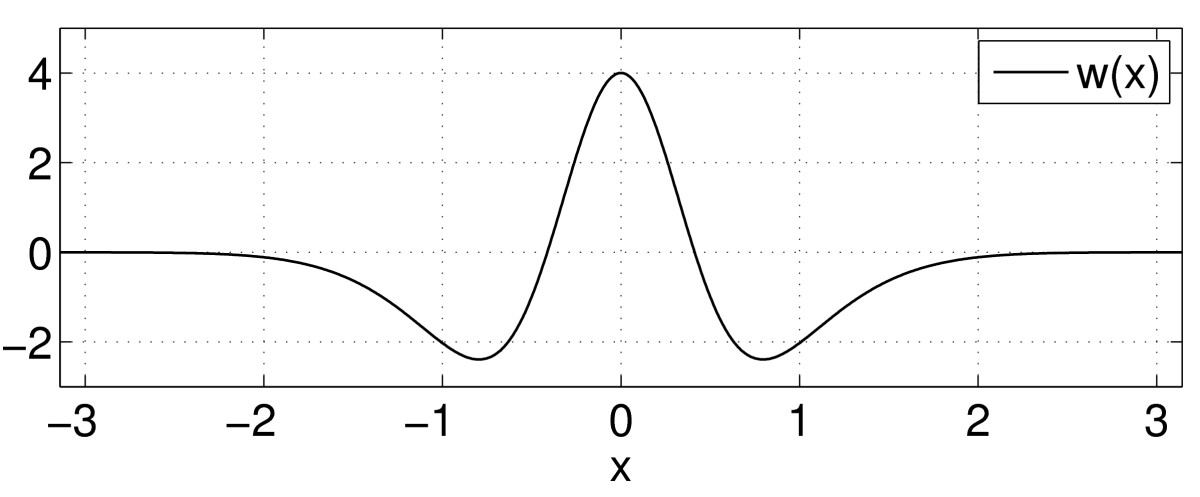
The connectivity function $w(x)=10\exp(-4x^{2})-6\exp(-x^{2})$

For concreteness, we will use the Mexican-hat connectivity function

12 $$w(x)=10\exp\left(-4x^{2}\right)-6\exp\left(-x^{2}\right);\;-\pi \leq x\leq \pi ,$$

as shown in Fig. 3. Stationary solutions of (10) satisfy

13 $$u(x)=\int_{-\pi }^{\pi }w(x-y)f\left(u(y)-h\right)\;dy,$$

and it is well known that in the limit β→∞ (i.e. the firing rate function *f* tends to the Heaviside) for a range of values of *h*, equation (13) supports two “bump” solutions, one of which is stable and the other unstable, under the dynamics of (10) [16, 20]. (Such bump solutions are thought to play a role in short term memory [24].) The two bumps annihilate one another in a saddle-node bifurcation as *h* is increased. Suppose we wanted to investigate the same phenomenon but for finite *β*, say β=20.

The first thing to note is that both (10) and (13) are invariant under translations, i.e. having found one solution, u(x) of (13), any translate, u(x+a), a∈R, is also a solution [25]. We want only one from this infinite family of solutions, so we need a way to select only one. A simple way to do this is to consider only even functions, i.e. functions for which u(−x)=u(x). Many steady states of equations like (10) are found to be even, but not all of them must be [26].

To represent u(x) we expand it as a Fourier series. Keeping in mind that it is an even function, we write

14 $$u(x,t)=\sum_{i=0}^{\infty }u_{i}(t)\cos(ix).$$

The function w(x) is also even, and we write it as a Fourier series

15 $$w(x)=\sum_{i=0}^{\infty }w_{i}\cos(ix),$$

where the $w_{i}$ can be found in the usual way: multiplying (15) by cos(jx) and integrating over [−π,π]. Using the identity cos(A−B)=cosAcosB+sinAsinB, substituting the two Fourier series above into (10) and equating like terms we find

16 $$\frac{du_{j}}{dt}=-u_{j}+w_{j}\int_{-\pi }^{\pi }\cos(jy)f\left(\sum_{i=0}^{\infty }u_{i}\cos(iy)-h\right)\;dy$$

for j=0,1,2,…, i.e. an infinite number of ordinary differential equations. The steady states of these satisfy

17 $$-u_{j}+w_{j}\int_{-\pi }^{\pi }\cos(jy)f\left(\sum_{i=0}^{\infty }u_{i}\cos(iy)-h\right)\;dy=0$$

for j=0,1,2,…. In practice we can only solve for a finite number of $u_{j}$, say *N*, so we solve

18 $$-u_{j}+w_{j}\int_{-\pi }^{\pi }\cos(jy)f\left(\sum_{i=0}^{N-1}u_{i}\cos(iy)-h\right)\;dy=0$$

for j=0,1,2,…,N−1. Note that if *w* is given exactly by a finite Fourier series, i.e. $w_{i}=0$ for $i>N_{F}$, then at steady state $u_{i}=0$ for $i>N_{F}$, and the truncation of (18) at $N-1=N_{F}$ will not introduce any errors, as noted by a number of authors [27–29].

We now show how to find and follow solutions of (18) as *h* is varied. Let **v** be the *N*-dimensional column vector with components $u_{0},u_{1},\ldots ,u_{N-1}$. The *N* scalar equations (18) can then be written in vector form as

19 F(v,h)=0,

where each component of this equation corresponds to one equation in (18), and $F:R^{N}\times R\to R^{N}$. For fixed *h*, say $h_{0}$, within some interval, we expect (19) to have several, isolated, solutions. One or more of these may be stable, and can be found by integrating (10) forward in time. Alternatively, a solution of (19) may be found using Newton’s method. Suppose one of these solutions is $v_{0}$, and we want to find another nearby solution, $\left(v_{1},h_{1}\right)$, which satisfies (19). The pseudo-arclength condition analogous to (3) is

20 $${\left(v_{1}-v_{0}\right)}^{T}{\dot{v}}_{0}+(h_{1}-h_{0}){\dot{h}}_{0}-\Delta s=0,$$

where a superscript “*T*” indicates transpose. This just states that $\left(v_{1},h_{1}\right)$ lies on the hyperplane perpendicular to the vector $\left({\dot{v}}_{0},{\dot{h}}_{0}\right)$, which at its closest point is a distance Δ*s* from $\left(v_{0},h_{0}\right)$. Analogously to (4), we see that the (N+1)-dimensional column vector

21 $$\left(\begin{array}{c} {\dot{v}}_{0}\\ {\dot{h}}_{0} \end{array}\right)$$

is the null vector of the N×(N+1) matrix $\left(F_{v}|F_{h}\right)$ where subscripts indicate partial derivatives (i.e. $F_{v}$ is the N×N Jacobian of *F* with respect to **v** and $F_{h}$ is a column vector of derivatives with respect to *h*) and these derivatives are evaluated at $\left(v_{0},h_{0}\right)$. Thus once the vector (21) has been found and normalised, it can be used in (20).

We solve $F(v_{1},h_{1})=0$ and (20) simultaneously using the analogue of (5), namely

22 $$\left(\begin{array}{c} v_{1}^{\left(i\right)}\\ h_{1}^{\left(i\right)} \end{array})=(\begin{array}{c} v_{1}^{\left(i-1\right)}\\ h_{1}^{\left(i-1\right)} \end{array})-J_{\left(i-1\right)}^{-1}(\begin{array}{c} F(v_{1}^{\left(i-1\right)},h_{1}^{\left(i-1\right)})\\ {\left(v_{1}^{\left(i-1\right)}-v_{0}\right)}^{T}{\dot{v}}_{0}+(h_{1}^{\left(i-1\right)}-h_{0}){\dot{\mu }}_{0}-\Delta s \end{array}\right)$$

for $i=1,2,\ldots ,N_{N}$, where

23 $$J_{\left(i\right)}=(\begin{array}{cc} F_{v} & F_{h}\\ {\dot{v}}_{0} & {\dot{h}}_{0} \end{array})$$

is the (N+1)×(N+1) Jacobian of the augmented system, and the partial derivatives are evaluated at $\left(v_{1}^{\left(i\right)},h_{1}^{\left(i\right)}\right)$. As above, we take $N_{N}$ Newton iterations and (assuming that (22) has converged) set $v_{1}=v_{1}^{\left(N_{N}\right)}$ and $h_{1}=h_{1}^{\left(N_{N}\right)}$. As an initial condition we can take $v_{1}^{\left(0\right)}=v_{0}+{\dot{v}}_{0}\Delta s$ and $h_{1}^{\left(0\right)}=h_{0}+{\dot{h}}_{0}\Delta s$. The stability of the fixed point $\left(v_{1},h_{1}\right)$ depends on the eigenvalues of $F_{v}$ evaluated at this point, and this matrix has already been calculated as the top left N×N block in the Jacobian *J*. We find the next solution $\left(v_{2},h_{2}\right)$ in exactly the same way as in Sect. 2, and can also use the approximation (8) if desired.

**Fig. 4:**
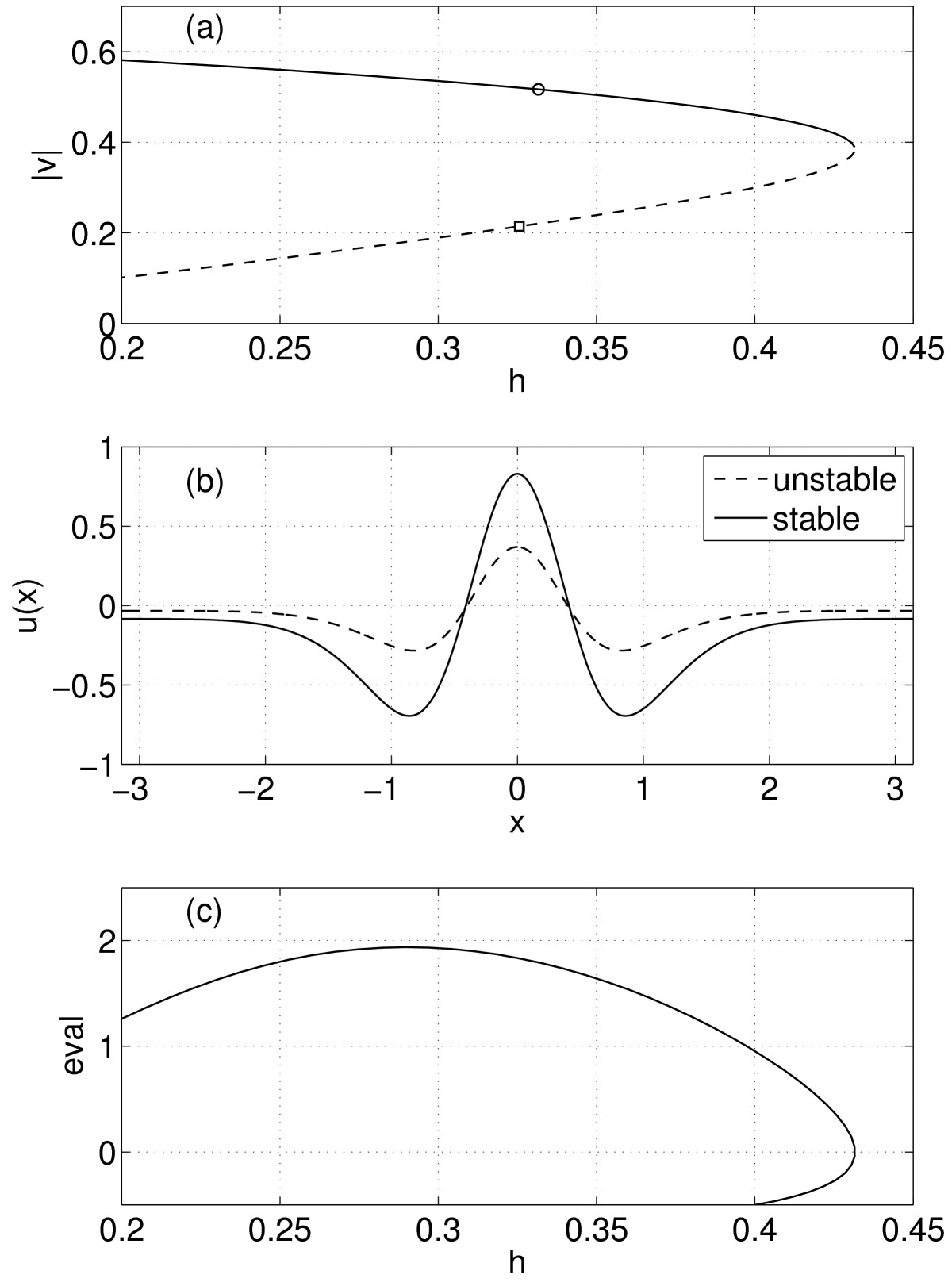
(**a**) Solutions of (19). The norm of *v* is plotted vertically; *solid curve* indicates stable solutions, *dashed* unstable. (**b**) Even solutions of (13) at the two points indicated in panel **(a)**. (**c**) Most positive eigenvalue associated with solutions of (19). Parameters: N=15, β=20

The results of following solutions of (19) as *h* is varied are shown in Fig. 4. In the top panel we see the stable and unstable solutions annihilating one another as *h* is increased, as expected. Representative solutions are shown in panel (b), and the most positive eigenvalue associated with the stability of solutions is shown in panel (c). Note that the zero eigenvalue associated with the translational invariance of solutions of (10) does not appear, as it has been removed by our choice of only considering even functions. Calculations were done with N=15, and the integrals in (18) were performed using the trapezoidal rule, which is extremely accurate on a periodic domain [30].

Several points should be made to end this section:

- An alternative to discretising (10) using Fourier series is to discretise the spatial domain [−π,π] directly, using a uniform grid. The integral in (10) can then be evaluated using the trapezoidal rule. Alternatively, since the integral is a convolution, it can be evaluated efficiently using the fast Fourier transform and multiplication in frequency space. Using this type of discretisation it is still straightforward to restrict to even functions. Essentially, one works with the function defined on only half of the domain ([−π,0]) and imposes evenness when necessary.
- As presented we can only find even solutions, and only determine stability with respect to perturbations that are also even. We will thus not detect any bifurcations leading to solutions which are not even.
- If we were interested in solutions that were not even, we could include sine terms in (14), substitute into (10), and derive the differential equations governing the evolution of their coefficients. We would then have to find some way of removing the translational invariance of solutions.
- The relationship between the symmetry of the system (i.e. its invariance with respect to group actions) and methods for choosing one from a continuous family of related solutions is discussed in more detail in [25, 31].
- Several other references dealing with continuation of high-dimensional problems in different contexts are [32–35].

We now consider moving patterns in one spatial dimension.

### 3.2 Moving Patterns

Let us again consider the model

24 $$\frac{\partial u(x,t)}{\partial t}=-u(x,t)+\int_{-\infty }^{\infty }w(x-y)f\left(u(y,t)-h\right)\;dy,$$

with *f* given by (11) but now with coupling function

25 $$w(x)=(1/2)e^{-|x|}.$$

Since $\int_{-\infty }^{\infty }w(x)\;dx=1$ we see that spatially uniform steady states of (24) satisfy

26 u=f(u−h).

**Fig. 5:**
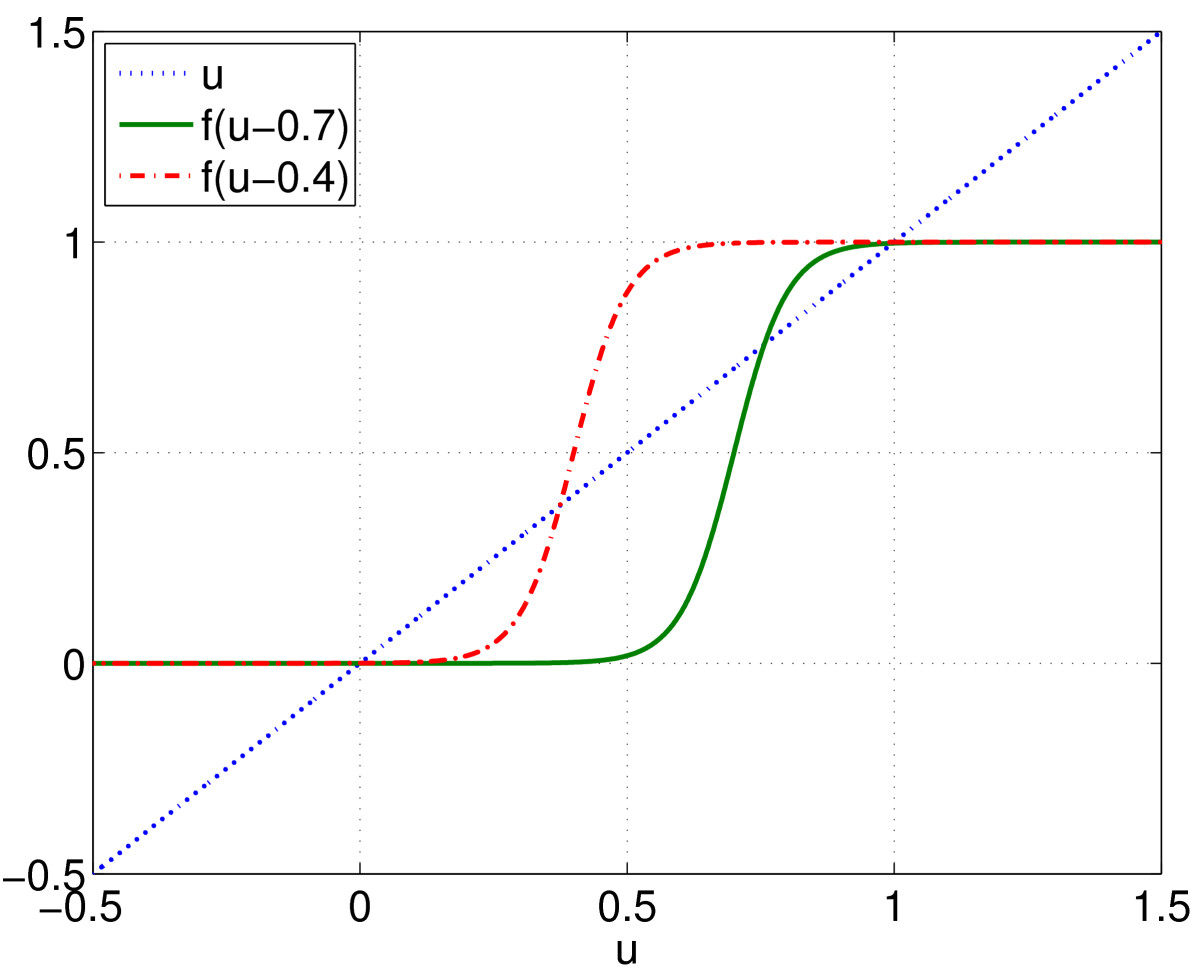
Solutions of (26) occur when the function f(u−h) intersects the diagonal, where *f* is given by (11) and β=20

**Fig. 6:**
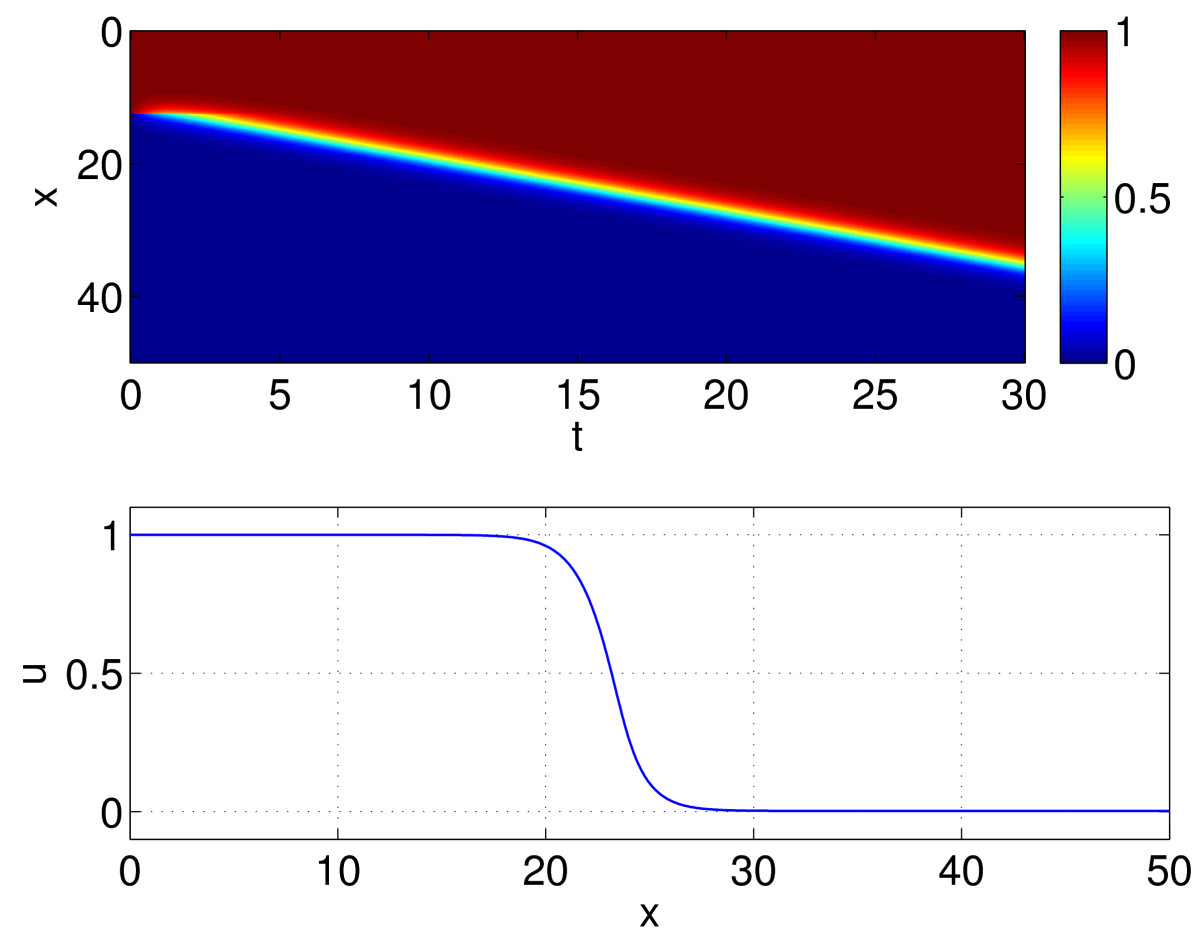
*Top*: space–time plot of a solution of (24)–(25), where the domain has been truncated to [0,50]. *Colour* indicates the value of *u*. *Bottom*: front at t=15. Parameters: β=20, h=0.3

Plotting *u* and f(u−h) on the same graph for several values of *h*, as in Fig. 5, we see that for moderate values of *h*, three such steady states exist, but if |h| is too large, only one exists. Let us choose a value of *h* such that three steady states exist, $u_{1}<u_{2}<u_{3}$, and initialise part of the domain at $u=u_{1}$ and the rest at $u=u_{3}$. Doing so we see the behaviour in Fig. 6, i.e. a front develops which connects these two steady states and moves with a constant speed in the direction of increasing *x*. We would like to find the shape of this front, and how its properties (speed, stability) depend on a parameter, say *h*.

To use the ideas above, we need to formulate an algebraic equation (or equations) satisfied by this front. We do this by moving to a travelling coordinate frame with the same speed as the front. Letting ξ=x−ct, equation (24) becomes

27 $$\frac{\partial u(\xi ,t)}{\partial t}=c\frac{\partial u(\xi ,t)}{\partial \xi }-u(\xi ,t)+\int_{-\infty }^{\infty }w(\xi -y)f\left(u(y,t)-h\right)\;dy.$$

If *c* is the speed of the front, then the front is stationary in the (ξ,t) coordinate system, i.e. it satisfies

28 $$0=c\frac{du}{d\xi }-u+\int_{-\infty }^{\infty }w(\xi -y)f\left(u(y)-h\right)\;dy.$$

A natural way to solve (28) is to truncate the domain and then discretise it using, say, *N* equally spaced grid points. The integral can be evaluated as in Sect. 3.1 and the spatial derivative using finite differences, or the fast Fourier transform [30]. However, imposing (28) at each grid point would give *N* equations, but there are N+1 unknowns, namely the value of *u* at each grid point, and the speed *c*. This is just a reflection of the fact that (24) is translationally invariant and thus there is a continuous family of front solutions satisfying (28), each differing from one another by a simple translation. As in Sect. 3.1 we need a way to choose just one of these. One simple way to do this is to impose the *pinning* condition

29 $$\int_{-\infty }^{\infty }(u-\hat{u}){\hat{u}}_{\xi }\;d\xi =0,$$

where $\hat{u}$ is a *template function*. Equation (29) is the result of minimising the $L^{2}$ norm of the difference between *u* and $\hat{u}$ [36, 37], so $\hat{u}$ should be somewhat similar to the solution one is trying to find. We can thus solve (28) and (29) simultaneously for *u* at each grid point and the speed *c*. Letting **v** be the (N+1)-dimensional column vector whose first *N* components are *u* at each of the *N* grid points, and whose (N+1)th component is *c*, equations (28) and (29) can be written as

30 F(v,h)=0,

where $F:R^{N+1}\times R\to R^{N+1}$. Once a pseudo-arclength condition like (20) has been appended, solutions of this set of N+2 equations can be followed just as in Sect. 3.1. To find the stability of a front found in this way at a particular value of *c*, we need to find all eigenvalues of the linearisation of (28) about the front. This linearisation appears as the top left N×N block in the Jacobian of the augmented system. Note that this linearisation has a zero eigenvalue with eigenvector equal to the (discretised) spatial derivative, ∂u/∂ξ. Stability of the front is determined by eigenvalues other than this one. (The stability of a wave in the original, i.e. non-discretised, system involves determining a continuous spectrum [38], and the discrete set of eigenvalues we find is an approximation to that.)

**Fig. 7:**
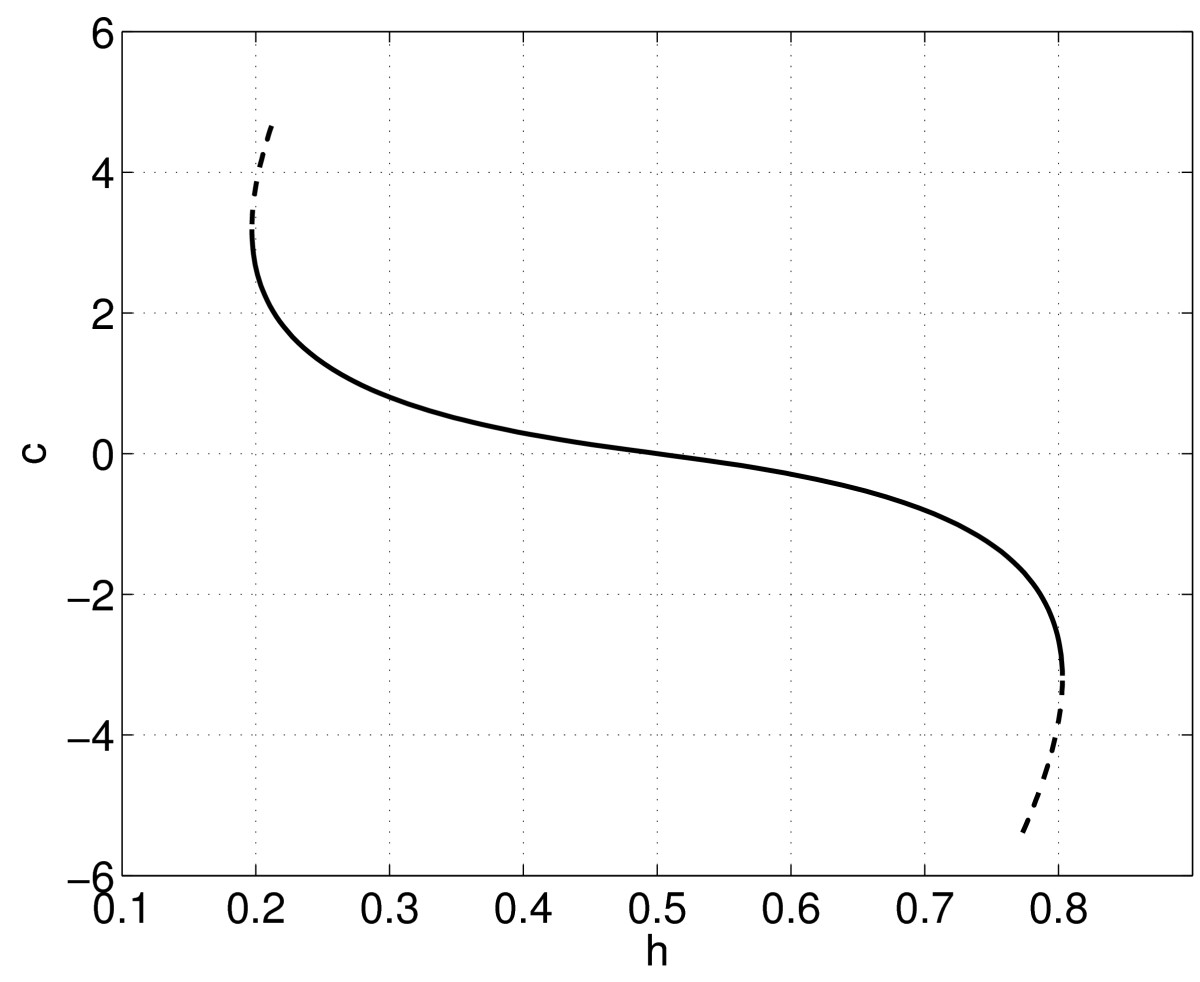
Speed of a front, *c*, as a function of *h*. *Solid*: stable, *dashed*: unstable. Parameters: β=20

The results of following the front as *h* is varied are shown in Fig. 7. We used 1,000 evenly spaced points over a domain of [0,50], and the template function was $\hat{u}(\xi )=0.5(1+\tanh(25-\xi ))$. We see that for a range of *h* values a stable front exists, and its speed may be positive, negative or zero, depending on the value of *h*. The stable front is destroyed in saddle-node bifurcations as *h* is varied too far from h=0.5 at the same values at which the spatially uniform steady states undergo saddle-node bifurcations, i.e. the stable front only exists when there are two stable spatially uniform steady states, which it connects. (The continuation was stopped after a number of points on each of the unstable branches were found.) Although we have only considered a front solution, the techniques described in this section can be used to follow other moving patterns such as “bumps” or pulses [27, 28, 37, 39, 40]. We now consider solutions in two spatial dimensions.

## 4 Two-Dimensional Models

**Fig. 8:**
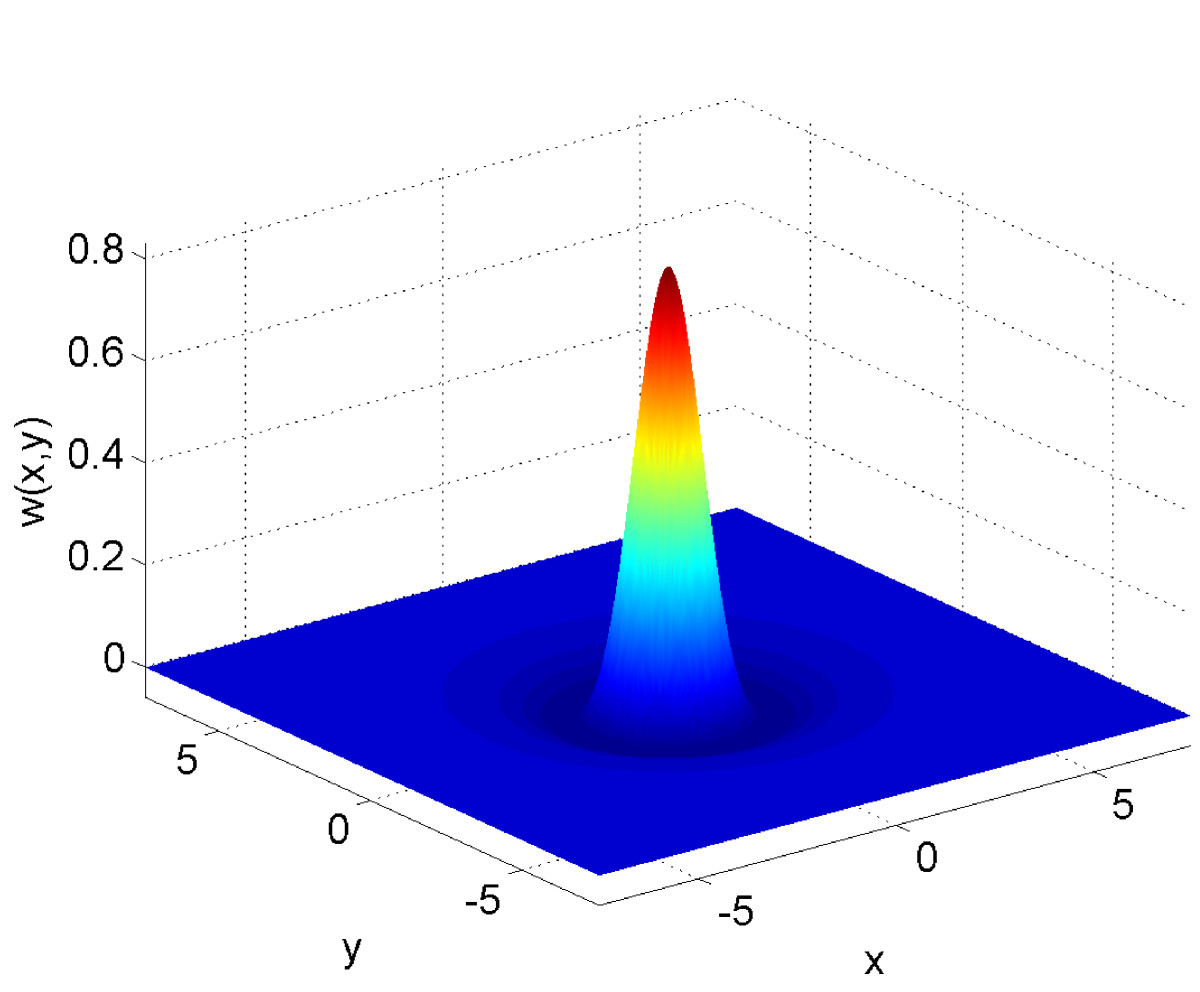
The Mexican-hat coupling function (33)

**Fig. 9:**
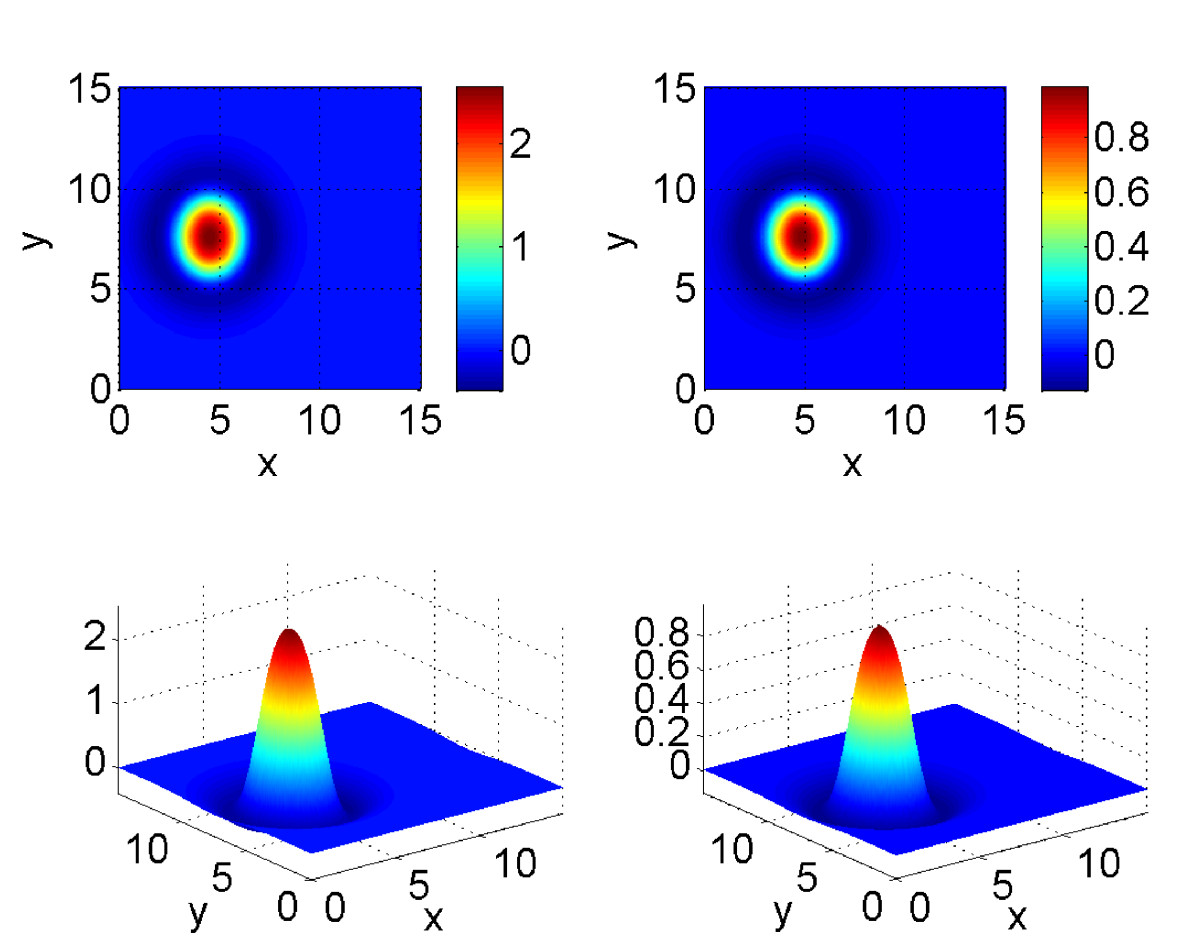
Leftward-moving bump solutions of (31)–(32). *Left column*: *u*, *right column*: *a*. Parameters: L=15, A=2, β=5, h=0.8, B=0.4, τ=3

We consider the model

31 $$\begin{array}{rl} \frac{\partial u(x,y,t)}{\partial t}= & A\int_{0}^{L}\int_{0}^{L}w\left(x-x^{\prime },y-y^{\prime }\right)f\left(u\left(x^{\prime },y^{\prime },t\right)-h\right)\;dx^{\prime }\;dy^{\prime }\\ -u(x,y,t)-a(x,y,t), \end{array}$$

32 $$\tau \frac{\partial a(x,y,t)}{\partial t}=Bu(x,y,t)-a(x,y,t),$$

defined on the square domain ${\left[0,L\right]}^{2}$ with periodic boundary conditions in both *x* and *y*. As well as the activity variable *u* we also have a “recovery” (or “adaptation”) variable *a*, which is driven by *u* with strength *B* on a timescale *τ*. The firing rate function is given by (11) and we use

33 $$w(x,y)=\exp\left(-\left(x^{2}+y^{2}\right)\right)-0.17\exp\left(-0.2\left(x^{2}+y^{2}\right)\right),$$

i.e. Mexican-hat connectivity, as shown in Fig. 8. When a=B=0, equations (31)–(32) are capable of supporting spatially localised “bump” solutions, and when the influence of *a* is strong enough (i.e. *B* is increased from zero) these bumps travel at a constant speed. An example is shown in Fig. 9. The bump is moving to the left (i.e. in the negative *x* direction) at a constant speed. Both *u* and *a* fields have a single peak, and the maximum of *a* is slightly behind that of *u*. We would like to find the dependence of this bump on various parameters in the model.

The first step is to move to a travelling coordinate frame in which the bump is stationary, as in Sect. 3.2. In principle the bump can move in any direction, but we specify that it is moving in the negative *x* direction. Letting ξ=x+ct, (31)–(32) become

34 $$\begin{array}{rl} \frac{\partial u(\xi ,y,t)}{\partial t}= & A\int_{0}^{L}\int_{0}^{L}w\left(\xi -x^{\prime },y-y^{\prime }\right)f\left(u\left(x^{\prime },y^{\prime },t\right)-h\right)\;dx^{\prime }\;dy^{\prime }\\ -u(\xi ,y,t)-a(\xi ,y,t)-c\frac{\partial u(\xi ,y,t)}{\partial \xi }, \end{array}$$

35 $$\tau \frac{\partial a(\xi ,y,t)}{\partial t}=Bu(\xi ,y,t)-a(\xi ,y,t)-c\tau \frac{\partial u(\xi ,y,t)}{\partial \xi },$$

and if *c* (>0) is the speed of the bump, it will be a stationary solution of (34)–(35), i.e. satisfy

36 $$\begin{array}{rl} 0= & A\int_{0}^{L}\int_{0}^{L}w\left(\xi -x^{\prime },y-y^{\prime }\right)f\left(u\left(x^{\prime },y^{\prime }\right)-h\right)\;dx^{\prime }\;dy^{\prime }\\ -u(\xi ,y)-a(\xi ,y)-c\frac{\partial u(\xi ,y)}{\partial \xi }, \end{array}$$

37 $$0=Bu(\xi ,y)-a(\xi ,y)-c\tau \frac{\partial u(\xi ,y)}{\partial \xi }.$$

Now any solution of (36)–(37) can be translated by an arbitrary amount in either the *ξ* or *y* direction, so we need to remove these degeneracies. We first impose the requirement that the solution be symmetric about y=L/2, i.e. u(ξ,y−L/2)=u(ξ,L/2−y) and similarly for *a*. We then impose a scalar condition which removes the invariance with respect to translations in the *ξ* direction. There are many ways to do this, but a simple and robust one is to specify that the value of *u* in the centre of the domain is equal to its average over *ξ* at y=L/2, i.e.

38 $$u(L/2,L/2)-\frac{1}{L}\int_{0}^{L}u(\xi ,L/2)\;d\xi =0.$$

(A condition like (29), imposed at y=L/2, could also be used. Note that since solutions of (36)–(37) are invariant under translations in *two* directions, we need to impose *two* conditions to remove these degeneracies.) We discretise the square domain with *N* equally spaced points in both directions. Imposing that (36) and (37) are satisfied at each of these $N^{2}$ points gives $2N^{2}$ equations, and combined with (38) we have $2N^{2}+1$ equations and the same number of unknowns. These equations can be written in the form

**Fig. 10:**
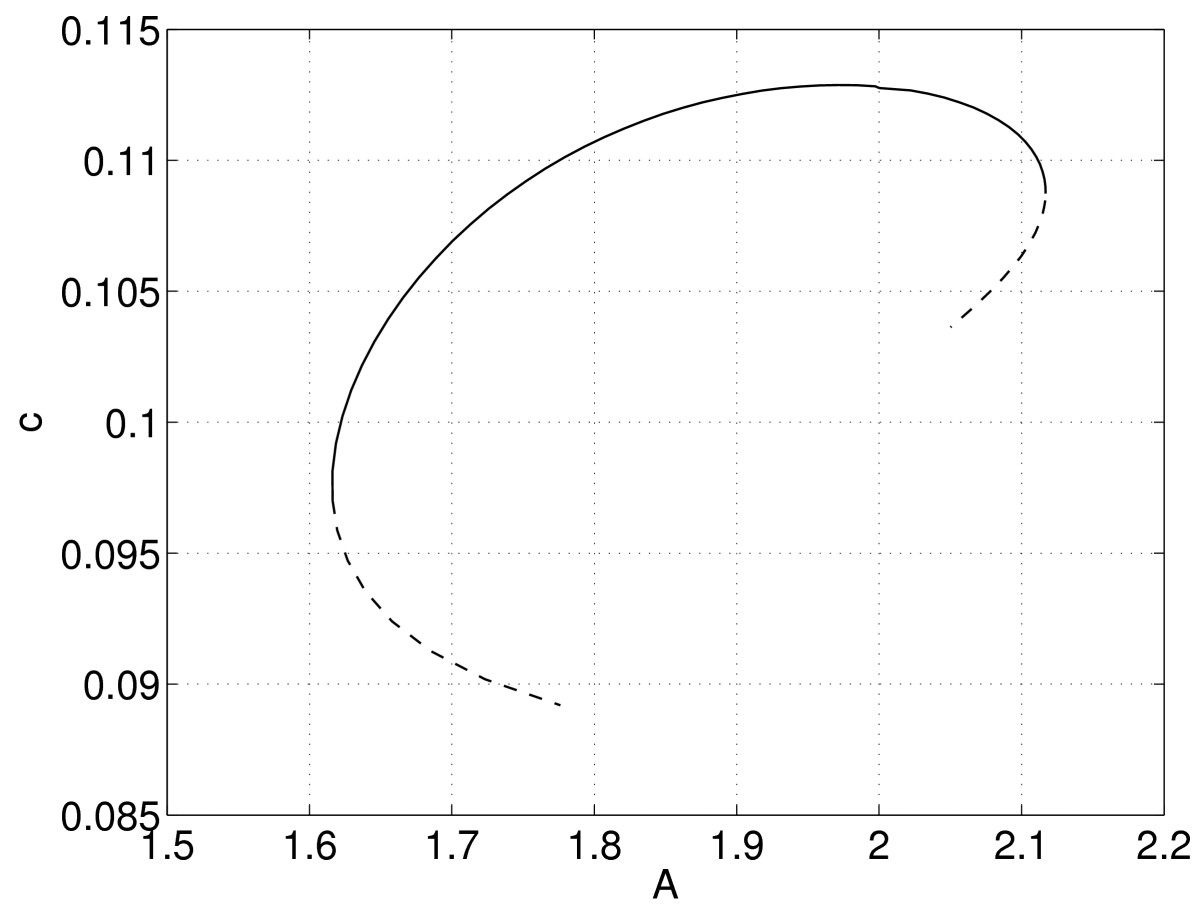
Speed of a two-dimensional bump, *c*, as a function of *A*. *Solid*: stable, *dashed*: unstable. Parameters: L=15, β=5, h=0.8, B=0.4, τ=3, N=256

39 F(v,λ)=0,

where $F:R^{2N^{2}+1}\times R\to R^{2N^{2}+1}$ and *λ* is one of the parameters of the system. Appending a pseudo-arclength condition to (39) one can follow solutions as *λ* is varied. (Note that (39) is typically a very large system of equations; see below.) Typical results are shown in Fig. 10 where we vary *A*. A stable bump is destroyed in saddle-node bifurcations as *A* is made either too large or too small. (The continuation was stopped after a number of points on each of the unstable branches were found.)

Note the following points:

- The convolution in (36) is evaluated using the two-dimensional Fast Fourier Transform (FFT), i.e. the FFT of (the discretisations of) both *w* and f(u−h) are taken, they are multiplied together, and then the inverse FFT is taken. Partial derivatives are evaluated using finite difference approximations, but could also be implemented using the FFT [30].
- Let us append a pseudo-arclength condition to (39) and define a vector $V\in R^{2N^{2}+2}$ by concatenating **v** and *λ*. The resulting set of equations can be written G(V)=0. Given a solution of (39), finding the next point along this curve amounts to solving G(V)=0 using Newton’s method, i.e. iterating
  40 $$V_{n+1}=V_{n}-J^{-1}G(V_{n});\;n=0,1,2,\ldots ,$$
  where *J* is the Jacobian of *G*, evaluated at $V_{n}$. For a reasonable discretisation, a large value of *N* is needed and hence *J* will be too big to store, let alone invert, so instead we write (40) as
  41 $$J\Delta_{n}=G(V_{n}),$$
  where $\Delta_{n}=V_{n}-V_{n+1}$. This is a linear equation for the unknown $\Delta_{n}$, but instead of solving it directly one can solve it iteratively, using for example the GMRES algorithm [41, 42]. Some implementations of the GMRES algorithm, e.g. that in Matlab, do not require the Jacobian *J* to be explicitly formed, only that one can evaluate the product of *J* with an arbitrary vector, ***ϕ***. This can be done for a general problem in a matrix-free way with one extra evaluation of *G*, using the finite difference approximation
  42 $$J\phi \approx \frac{G(V_{n}+\epsilon \phi )-G(V_{n})}{\epsilon },\;0<\epsilon \ll 1.$$
  Similarly, we need the eigenvalues of *J*, or at least a few with the largest real part, to determine stability. The Matlab function eigs does not need *J*, only its product with an arbitrary vector, which can be implemented as above.
  Note that for the particular problem considered here, the product of *J* with an arbitrary vector can be calculated exactly without the need for the approximation (42), as explained by Rankin et al. [43]. These authors used GMRES to follow stationary solutions of neural field equations in two dimensions, but the results here may be the first for travelling solutions.
- As well as travelling bumps [44], patterns that appear in two spatial dimensions include stationary groups of bumps [19, 37, 43], “breathing” bumps [45], rings and rotating groups of bumps [46], waves [47], spirals [48, 49] and target patterns [45, 50]. While stationary patterns and those that propagate at a constant velocity (either translational or rotational) can be dealt with using the ideas in this section, patterns such as breathing bumps and target waves are intrinsically periodic in time, and thus must be dealt with using slightly different techniques.
- We have evaluated the double integral in (31) directly using fast Fourier transforms, but some early progress on two-dimensional neural fields was made using other Fourier techniques [19, 48], and see [51]. For example, suppose that the Fourier transform of *w* was
  43 $$\frac{1}{s^{4}+s^{2}+1},$$
  where $s^{2}=k_{x}^{2}+k_{y}^{2}$ and $k_{x}$ and $k_{y}$ are the two transform variables. Taking the two-dimensional Fourier transform of (31) we obtain
  44 $$\frac{\partial \hat{u}}{\partial t}+\hat{u}+\hat{a}=\frac{A\hat{f}}{s^{4}+s^{2}+1},$$
  where the hat indicates the Fourier transform. Multiplying (44) by $s^{4}+s^{2}+1$, and taking the inverse Fourier transform, using a Fourier transform identity we obtain
  45 $$\left(\nabla^{4}-\nabla^{2}+1\right)\left(\frac{\partial u(x,y,t)}{\partial t}+u(x,y,t)+a(x,y,t)\right)=Af\left(u(x,y,t)-h\right),$$
  which is formally equivalent to (31) but only involves derivatives. The advantage of this formulation is that solutions of (36)–(37) satisfy
  46 $$\left(\nabla^{4}-\nabla^{2}+1\right)\left(c\frac{\partial u}{\partial \xi }+u+a\right)=Af(u-h),$$
  47 $$c\tau \frac{\partial u}{\partial \xi }=Bu-a,$$
  which only involve derivatives. Finite difference approximations to these derivatives can then be implemented using sparse matrices, thus removing the need to store and manipulate large full matrices. This idea has subsequently been used by several other groups [47, 50].
  Using this method, the coupling function is assumed to be a function of only distance in two dimensions and is given by
  48 $$w(r)=\int_{0}^{\infty }sJ_{0}(rs)\hat{w}(s)\;ds,$$
  where $J_{0}$ is the Bessel function of the first kind of order zero and $\hat{w}(s)$ is the Fourier transform of *w* (in the case above, $\hat{w}(s)=1/(s^{4}+s^{2}+1)$).

We now discuss a number of extensions to the ideas presented here.

## 5 Extensions

### 5.1 Delays

We have considered differential equations where the derivatives depend on only the values of the variables at the present time. However, delays are ubiquitous in neural systems [52–56] (and elsewhere) so the study of delay differential equations naturally arises. Such systems can be numerically integrated using, for example, Matlab’s dde23, but following periodic orbits and determining the stability of fixed points is much more involved than for non-delayed systems, due to the infinite-dimensional nature of the problem, even for a scalar equation. The software package DDE-BIFTOOL [11] is useful for performing such calculations, and also see [57].

### 5.2 Global Bifurcations

Following solutions using pseudo-arclength continuation and determining their stability via linearisation about them will only detect local bifurcations such as saddle-node and Hopf. However, global bifurcations can also play a role in determining the dynamics of a system [58–60]. They also provide another way of viewing spatially localised “bumps,” or fronts. As an example, consider (28), writing *x* instead of *ξ*. Using the fact that the coupling function (25) is the Green function of $\left(1-d^{2}/dx^{2}\right)$, acting on (28) with this operator gives

49 $$0=\left(1-\frac{d^{2}}{dx^{2}}\right)\left(c\frac{d}{dx}-1\right)u+f\left(u(x)-h\right),$$

i.e.

50 $$cu^{\prime \prime \prime }-u^{\prime \prime }-cu^{\prime }+u=f(u-h),$$

where the primes indicate derivative with respect to space. (Another way to derive (49) is to take Fourier transforms of (28), use the fact that the transform of *w* is $1/(1+k^{2})$ where *k* is the transform variable, then rearrange and take the inverse Fourier transform [22].) This ordinary differential equation has fixed points which satisfy u=f(u−h) and the front in Fig. 6 is a heteroclinic connection between two of these fixed points. Now (50) can be written

51 $$u^{\prime }=v,$$

52 $$v^{\prime }=z,$$

53 $$z^{\prime }=\frac{f(u-h)}{c}-\frac{u}{c}+v+\frac{z}{c},$$

with Jacobian at the fixed point $\left(u,v,z)=(\overset{?}{u},0,0\right)$ given by

54 $$J=(\begin{array}{ccc} 0 & 1 & 0\\ 0 & 0 & 1\\ \frac{f^{\prime }(\overset{?}{u}-h)-1}{c} & 1 & \frac{1}{c} \end{array}).$$

The eigenvalues of this, *λ*, satisfy

55 $$\lambda^{3}-\lambda^{2}/c-\lambda +\left(1-f^{\prime }(\overset{?}{u}-h)\right)/c=0.$$

For h≈0.5, $f^{\prime }(\overset{?}{u}-h)\approx 0$, where $\overset{?}{u}$ is either the lower fixed point, $u_{1}$, or the upper one, $u_{3}$ (see Fig. 5 and Sect. 3.2) and thus (55) can be written approximately as

56 (λ−1)(λ+1)(λ−1/c)=0,

i.e. both fixed points have a two-dimensional unstable manifold and one-dimensional stable manifold (for c>0). A heteroclinic connection between the fixed points occurs when the unstable manifold of one intersects the stable manifold of the other, which is a codimension-one event for this system, i.e. it will generically occur at isolated values of the parameter *c*. These values are those shown in Fig. 7. They can be found by “shooting”: numerically integrating (51)–(53) backwards using an initial condition on the stable manifold of one fixed point, and varying *c* until this trajectory intersects the unstable manifold of the other fixed point. If *c* is negative the dimensions of the stable and unstable manifolds are interchanged, but the argument above still applies.

Notes:

- In the same way that a front can be viewed as a heteroclinic connection between two fixed points, a spatially localised pulse can be viewed as a homoclinic orbit to a fixed point. This applies whether the pulse is stationary [18, 61, 62] or moving [39] (in which case the speed appears as a parameter, as above).
- Software for the continuation of homoclinic and heteroclinic orbits exists [10, 63].
- The conversion of an integral equation like (28) to a differential equation via Fourier transform has been used by a number of authors [18, 61, 62, 64]. For this technique to work, the Fourier transform of the coupling function should be a rational function of the square of the transform variable.
- The resulting differential equations sometimes have additional struction (they are Hamiltonian, for example) and this can be exploited in their analysis [18, 61].
- Solutions which are periodic in space may also be of interest, and it may be easier to find them by considering periodic solutions of a differential equation of the form (50) rather than the equivalent integral equation (28).

### 5.3 Following Bifurcations

So far we have followed solutions of algebraic equations as a single parameter has varied. Local bifurcations occur at isolated values of this parameter. However, it is often more informative to *follow* these bifurcations as a second parameter is varied. To demonstrate this we return to the problem in Sect. 3.1, i.e. we look for stationary solutions of (10) where *f* is given by (11) but the coupling function is

57 $$w(x)=10\exp\left(-4x^{2}\right)-B\exp\left(-x^{2}\right).$$

The analysis in Sect. 3.1 corresponds to the case B=6, but we would like to know the effect of varying *B* on the range of values of *h* for which stable solutions exist (see Fig. 4). We proceed as in Sect. 3.1 but the coefficients $w_{i}$ are now functions of *B*, i.e. we solve

58 $$-u_{j}+w_{j}(B)\int_{-\pi }^{\pi }\cos(jy)f\left(\sum_{i=0}^{N-1}u_{i}\cos(iy)-h\right)\;dy=0$$

for j=0,1,2,…,N−1. This set of equations can be written compactly as

59 F(v,h,B)=0,

as before, where $F:R^{N}\times R\times R\to R^{N}$. For a given *h* and *B*, a solution of (59) is a stationary solution of (10). However, for a fixed *B* we would like to find the value of *h* at which the saddle-node bifurcation occurs, as in Fig. 4. At this point the Jacobian of *F* has a zero eigenvalue. The eigenvector $\phi \in R^{N}$ corresponding to this eigenvalue then satisfies

60 Jϕ=0,

where *J* is the N×N Jacobian. Now ***ϕ*** is unique up to a scaling of its magnitude so to choose a particular ***ϕ*** we impose

61 $$\phi^{T}\phi -1=0,$$

i.e. that ***ϕ*** has unit length. Thus for a fixed *B*, the value of *h* at which the saddle-node bifurcation occurs (and the solution at that point, **v**) satisfy

62 F(v,h,B)=0,

63 Jϕ=0,

64 $$\phi^{T}\phi -1=0.$$

This is a set of 2N+1 equations in 2N+1 unknowns (the *N* components of **v**, the *N* components of ***ϕ***, and *h*). Note that *J* depends on **v**, *h* and *B*, and that the product of *J* and ***ϕ*** can be found easily using the method discussed in Sect. 4. Concatenating **v**, ***ϕ*** and *h* to form the (2N+1)-dimensional vector **W**, equations (62)–(64) can be written

**Fig. 11:**
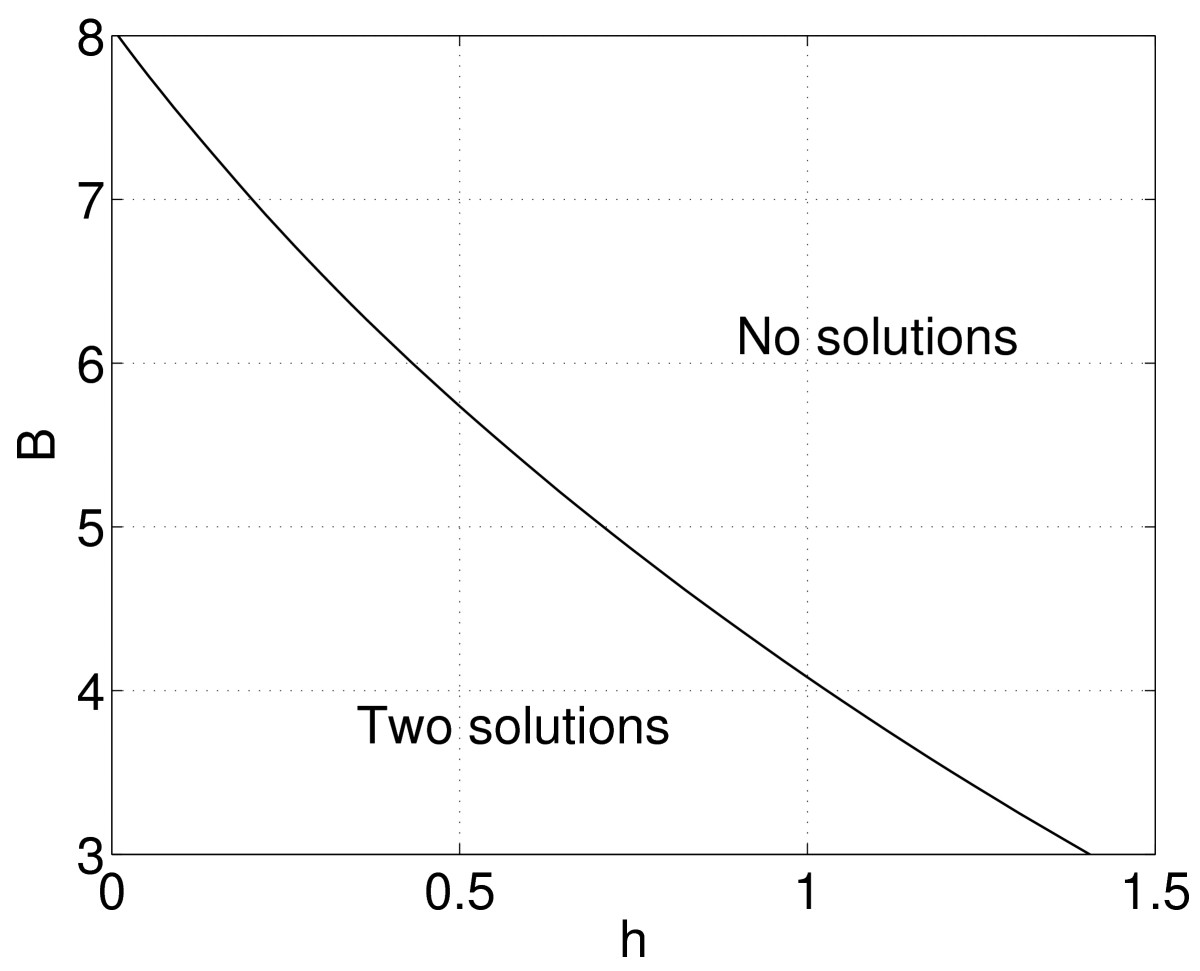
Curve of saddle-node bifurcations of stationary single-bump solutions of (10), where the coupling function is (57). A stable and unstable pair exist to the left of this curve. Figure 4(a) is a horizontal “slice” through this figure at B=6. Parameters: N=15, β=20

65 H(W,B)=0,

where $H:R^{2N+1}\times R\to R^{2N+1}$. Solutions of (65) can be followed as *B* is varied in the usual way, and plotting the values of *B* found versus the corresponding element of the **W** s, the curve of saddle-node bifurcations can be traced out in the (h,B) plane. The results of doing this are shown in Fig. 11. We see that as *B* is decreased, the value of *h* at which the saddle-node bifurcation occurs increases and vice versa.

Although we have not shown any here, Hopf bifurcations can also occur in neural field models, leading to oscillatory behaviour [20, 22, 65, 66]. They are characterised by a pair of complex conjugate eigenvalues of the Jacobian passing through the imaginary axis. Curves of Hopf bifurcations can be followed as two parameters are varied in a similar way to that explained above for saddle-node bifurcations, the main difference being that in the simplest formulation there are O(3N) equations to solve rather than O(2N), as both the real and the imaginary parts of the corresponding equations have to be solved [8, 67]. Note that more sophisticated algorithms can reduce the number of equations to be solved when following both saddle-node and Hopf bifurcations [4].

### 5.4 Maps

Pseudo-arclength continuation is a method for following solutions of algebraic equations as a parameter is varied. In this paper we have used algebraic equations which define stationary solutions of differential equations, in either a stationary or uniformly travelling coordinate frame. However, for some dynamical systems we may be interested in fixed points of a discrete-time map which may correspond to, for example, periodic orbits of an underlying system [68–70] (and see below). The equations defining these fixed points are also algebraic and can thus be treated using the same methods as discussed above. Note that the criteria for stability of a fixed point of a differential equation is different from that of a fixed point of a map [58–60].

### 5.5 Periodic Orbits

We have only considered stationary solutions of differential equations, but many differential equations have solutions which are periodic in time, arising from, for example, a Hopf bifurcation [58, 59]. Periodic forcing, in either time or space [71, 72], can also generate solutions which are periodic in time or space, respectively. Finding and following periodic orbits can be done using pseudo-arclength continuation. The main idea is as before: construct a set of algebraic equations which are satisfied by the periodic orbit. Such an orbit is represented in a finite-dimensional way using, for example, a finite Fourier series expansion, or more commonly and efficiently, a piecewise polynomial function [59, 73] for each variable. This representation of the orbit is then substituted into the governing differential equations, giving a set of algebraic equations that must be satisfied. For autonomous differential equations (i.e. ones which do not explicitly depend on time) there is an invariance with respect to time shifts, in the same way that we saw invariance with respect to spatial shifts in Sect. 3. This invariance can be removed in the same way, by using a scalar “phase” condition to select one from a continuous family [73].

**Fig. 12:**
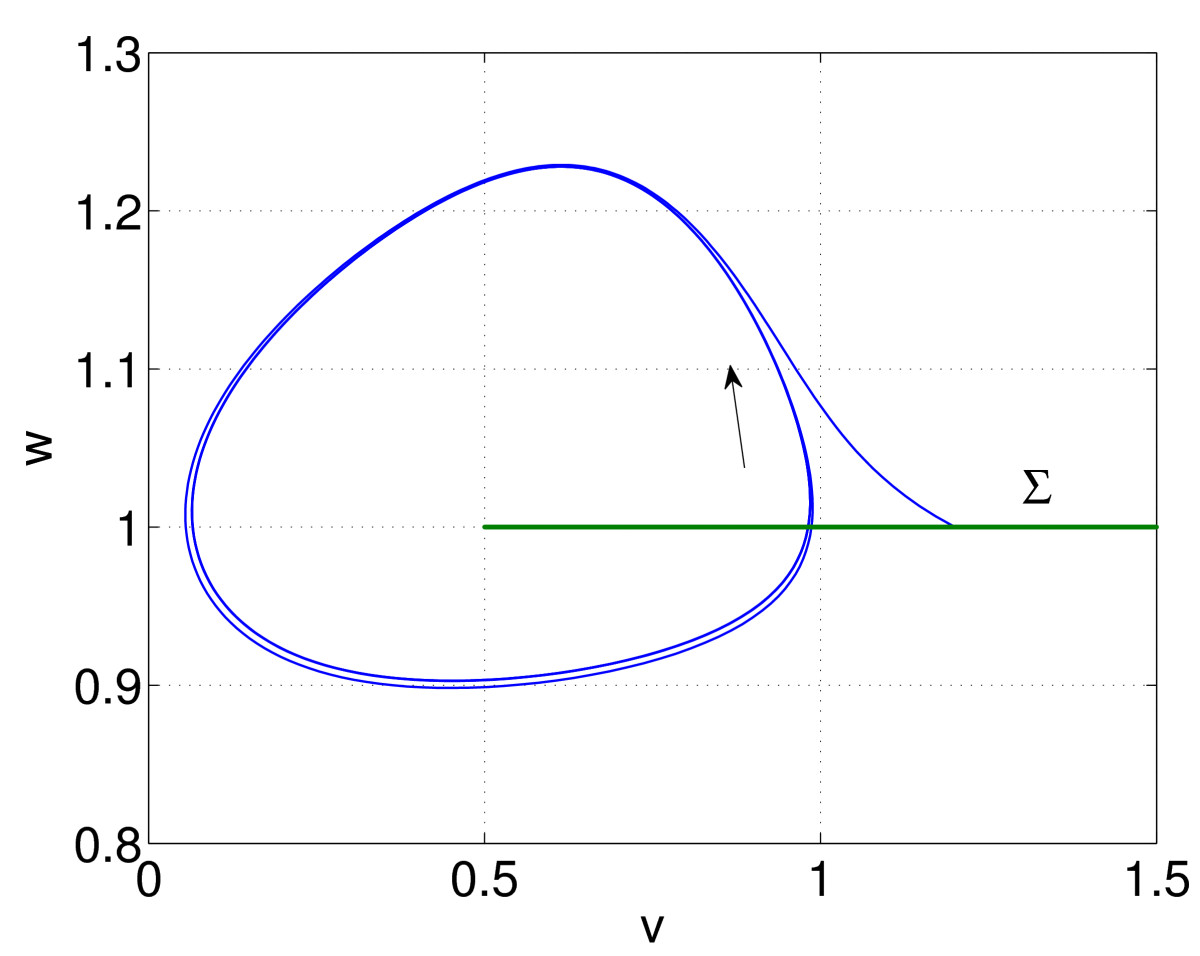
Finding a periodic orbit of (66)–(67) using a Poincaré section, *Σ*. The *arrow* indicates the direction of motion. See text for details

An alternative method for finding periodic orbits is to put a Poincaré section in the phase space in such a way as to intersect the periodic orbit, which then becomes a fixed point of a map defined from the section to itself. As an example, consider the FitzHugh–Nagumo equations [74]

66 $$\frac{dv}{dt}=\frac{v(v+0.1)(1-v)-w+1}{0.1},$$

67 $$\frac{dw}{dt}=v-0.5w,$$

which have a stable periodic orbit. Figure 12 shows the stable periodic orbit and a transient. We define the Poincaré section

68 Σ={(v,w):v>0.5,w=1},

and choose an initial condition on it, $\left(v,w)=(V_{0},1\right)$. ($V_{0}=1.2$ in Fig. 12.) Now integrate (66)–(67) using this initial condition until the solution is again in *Σ*, at $\left(v,w)=(V_{1},1\right)$. Repeat, using this initial condition, and thus generate a sequence $V_{0}$, $V_{1}$, $V_{2}\ldots$, which are iterates of a map:

69 $$V_{i}=\psi (V_{i-1}),\;i=1,2,\ldots$$

The periodic orbit in Fig. 12 is a fixed point of this map, defined by $V^{*}=\psi (V^{*})$, an algebraic equation whose solutions can be followed as parameters are varied. Note that *ψ* is *defined* using numerical integration of a set of differential equations, and this integration must be done accurately for the technique to be successful [68]. This method will find an unstable periodic orbit, as long as the initial condition is close enough to it. A modification of this approach involves “multiple shooting,” where several Poincaré sections are put in phase space, and one successively integrates from one to the next and concatenates the results [59, 75].

This technique, of using numerical integration of the underlying system over short time intervals to find both stable and unstable objects, is an example of bifurcation analysis using timesteppers [76]. It forms the basis of the “equation-free” approach, which we now discuss.

### 5.6 Equation-Free

When studying systems such as (1) it is often assumed that one can explicitly and quickly evaluate g(u;μ) using a subroutine or by calling a Matlab “function file,” for example. However, there is no requirement that g(u;μ) be explicitly specified and as long as, given *u* and *μ*, a reasonably accurate estimate of g(u;μ) can be obtained, one can use the ideas presented above. This idea forms part of the “equation-free” approach to studying complex multiscale systems [77–79]. We now briefly summarise some of the relevant ideas.

Suppose that we have a well-defined dynamical system

70 $$\frac{dv}{dt}=\phi (v;\mu ),$$

where $v\in R^{n}$ and 1≪n. The components of **v** could be all of the variables associated with a network of Hodgkin–Huxley-type neurons, or the positions and velocities of a number of interacting particles for example. We refer to (70) as the “microscopic” or “fine-scale” description of our model. Suppose also that we believe that (70) can be effectively described by the dynamical system

71 $$\frac{dV}{dt}=\Phi (V;\mu ),$$

where $V\in R^{m}$ and m≪n. Determining that this is the case—and what the variable **V** actually is—is a complex topic that we will not go into here, but at its simplest, a particular component of **V** could be the average of some components of **v**, for example. By “effectively described” we mean that a bifurcation analysis, or numerical integration, of (71) would give similar results as performing these operations on (70), with any differences being easily explained and unimportant. We refer to (71) as the “macroscopic” or “coarse” description of our model. We would like to perform bifurcation analysis of (71), but we do not have an explicit expression for *Φ*. To evaluate Φ(V;μ) for particular **V** and *μ* we need two operators which map between **v** and **V**. The first is a *lifting* operator $L:R^{m}\to R^{n}$ such that L(V)=v. We also need a *restricting* operator $R:R^{n}\to R^{m}$ such that R(v)=V. These operators must satisfy the consistency condition R(L(V))=V. Since *L* maps from a low-dimensional space to a high-dimensional one it is often not unique.

To evaluate $\Phi (V_{0};\mu_{0})$ we first lift $V_{0}$ to produce $v_{0}$, where $L(V_{0})=v_{0}$. We then initialise the fine-scale model (70) with the initial condition $v_{0}$ (and $\mu =\mu_{0}$) and simulate (70) for a short amount of time, of duration say Δ, resulting in the state v(Δ). Restricting this gives an estimate of V(Δ), i.e. V(Δ)=R(v(Δ)). We thus have

72 $$\frac{V(\Delta )-V_{0}}{\Delta }\approx \frac{dV}{dt}{|}_{V=V_{0}}=\Phi (V_{0};\mu_{0}).$$

In other words, we estimate Φ(V;μ) by running a short “burst” of the microscopic system, suitably initialised, and restricting the result. Since there are normally many different $v_{0}$ consistent with a particular $V_{0}$, running many bursts with the different $v_{0}$ and then averaging often results in a better estimate of $\Phi (V_{0};\mu_{0})$—this is ideally done in parallel on multiple processors. Thus in principle we can evaluate Φ(V;μ) for any **V** and *μ*, and this is all we need to perform bifurcation analysis (or numerical integration) of (71). Note that **V** can be approximately stationary even though **v** is not (if **V** is average firing rate of a network, and **v** contains voltages of neurons in the network, for example) so when finding “fixed points” of *Φ*, one may need to relax the criteria for determining when Φ=0.

Often in the equation-free approach “the devil is in the details,” and a number of applications in computational neuroscience are demonstrated in [80–82]. As shown in these papers, the choice of **V** can often be done semi-automatically using techniques from data-mining. (A long simulation of (70) is done, and the results mined to find whether there is a low-dimensional parametrisation of the data set. If so, these parameters form the components of **V**.) Note that while we have written the microscopic model (70) as a deterministic differential equation, it could equally well be a stochastic differential equation, or even a dynamical system in which both time and state are discrete [79, 83]. An interesting generalisation of the method presented here was shown in [84], where the analogue of the function *Φ* was evaluated *experimentally* in real time, rather than by running a computer simulation.

## 6 Conclusion

Numerical continuation is a powerful technique, allowing one to follow solutions of sets of algebraic equations as a parameter is varied. We have given an introduction to this technique, discussed its use in the investigation of various models arising in computational neuroscience, and demonstrated its use with a number of examples. The technique is general, but we have concentrated on high-dimensional systems which arise as discretisations of neural field models. By suitable modification the technique can be used to follow bifurcations as two parameters are varied, and to follow solutions which are periodic in time, or uniformly translating or rotating. Its use in high-dimensional systems has been restricted in the past by issues of memory and computational time, but with cheaper memory and faster processors continuing to be produced, we expect the technique to continue to be useful for the investigation of complex, high-dimensional dynamical systems.

## Authors’ original submitted files for images

Below are the links to the authors’ original submitted files for images. Authors’ original file for figure 1 Authors’ original file for figure 2 Authors’ original file for figure 3 Authors’ original file for figure 4 Authors’ original file for figure 5 Authors’ original file for figure 6 Authors’ original file for figure 7 Authors’ original file for figure 8 Authors’ original file for figure 9 Authors’ original file for figure 10 Authors’ original file for figure 11 Authors’ original file for figure 12 Authors’ original file for figure 13
