# Supplementary figures and images for: Numerical Bifurcation Theory for High-Dimensional Neural Models
Source: J Math Neurosci. 2014 Jul 25;4:13. doi: 10.1186/2190-8567-4-13 (PMC7224244; doi:10.1186/2190-8567-4-13)

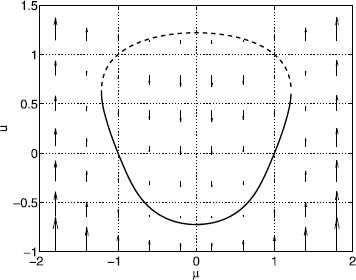

Supplement: Supplementary file 1 — Authors’ original file for figure 1 [file 13408_2014_58_MOESM1_ESM.gif]

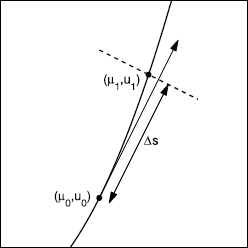

Supplement: Supplementary file 2 — Authors’ original file for figure 2 [file 13408_2014_58_MOESM2_ESM.gif]

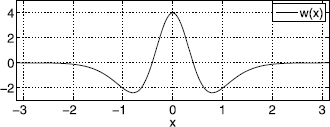

Supplement: Supplementary file 3 — Authors’ original file for figure 3 [file 13408_2014_58_MOESM3_ESM.gif]

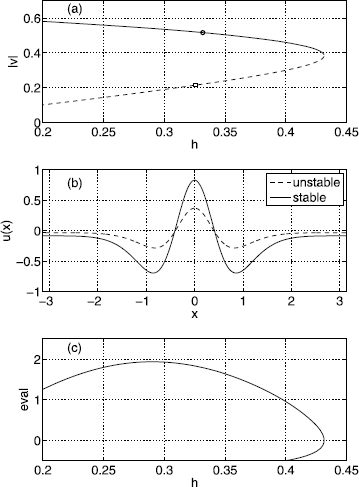

Supplement: Supplementary file 4 — Authors’ original file for figure 4 [file 13408_2014_58_MOESM4_ESM.gif]

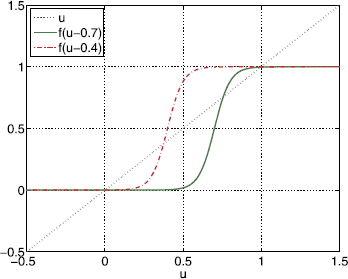

Supplement: Supplementary file 5 — Authors’ original file for figure 5 [file 13408_2014_58_MOESM5_ESM.gif]

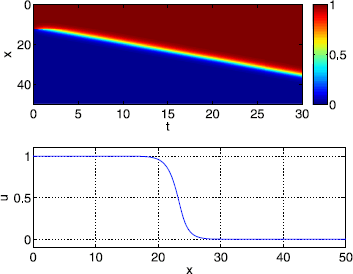

Supplement: Supplementary file 6 — Authors’ original file for figure 6 [file 13408_2014_58_MOESM6_ESM.gif]

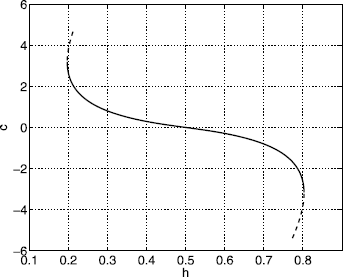

Supplement: Supplementary file 7 — Authors’ original file for figure 7 [file 13408_2014_58_MOESM7_ESM.gif]

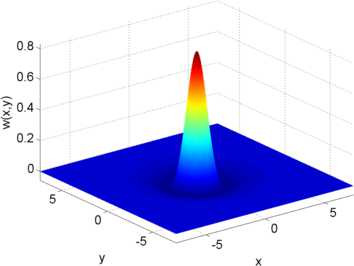

Supplement: Supplementary file 8 — Authors’ original file for figure 8 [file 13408_2014_58_MOESM8_ESM.jpg]

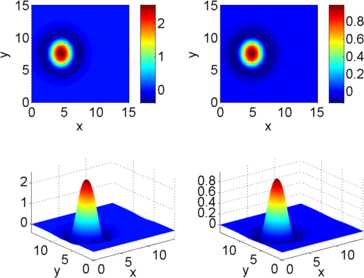

Supplement: Supplementary file 9 — Authors’ original file for figure 9 [file 13408_2014_58_MOESM9_ESM.jpg]

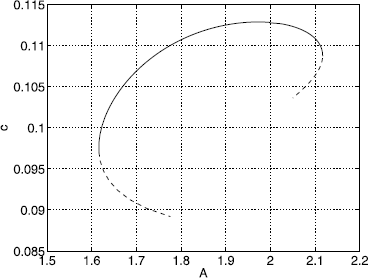

Supplement: Supplementary file 10 — Authors’ original file for figure 10 [file 13408_2014_58_MOESM10_ESM.gif]

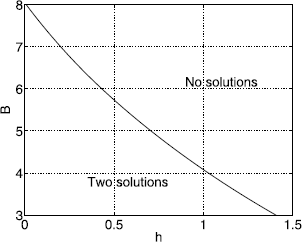

Supplement: Supplementary file 11 — Authors’ original file for figure 11 [file 13408_2014_58_MOESM11_ESM.gif]

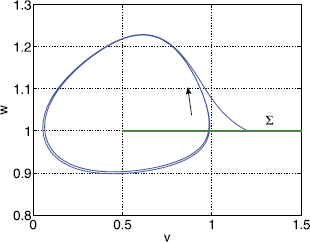

Supplement: Supplementary file 12 — Authors’ original file for figure 12 [file 13408_2014_58_MOESM12_ESM.gif]
